# Dietary Tryptophan Allocation in Depression: Serotonin–Kynurenine Balance, Microbial Indole Pathways, and Inflammatory Phenotypes

**DOI:** 10.3390/nu18162716

**Published:** 2026-08-20

**Authors:** Bernard Kordas

**Affiliations:** Department of Human Physiology and Pathophysiology, School of Medicine, Collegium Medicum, University of Warmia and Mazury, 10-082 Olsztyn, Poland; bernard.kordas@uwm.edu.pl

**Keywords:** major depressive disorder, tryptophan, kynurenine, serotonin, gastrointestinal microbiome, indoles, inflammation, metabolomics, dietary supplements

## Abstract

Depression is defined by clinical symptoms, but its biology varies considerably among patients. In this review, dietary tryptophan allocation describes the routes taken by tryptophan after intestinal absorption. Some is incorporated into proteins. Some enters serotonin and melatonin synthesis or the kynurenine pathway, while gut bacteria convert another fraction into indole compounds. Brain availability also depends on the circulating free pool and competition with other large neutral amino acids. This term does not imply a new biochemical pathway. It allows these known processes to be considered in relation to inflammation, metabolism, the gut microbiota, medication use, and current disease state. Recent meta-analyses indicate that peripheral tryptophan is lower in depression. They do not show a consistent increase in the kynurenine-to-tryptophan ratio, and findings from cerebrospinal fluid vary between studies. Human multiomics studies have associated microbial and metabolite profiles with cognition and response to treatment, although the evidence remains largely correlational. Changes in kynurenine, 3-hydroxykynurenine, and quinolinic acid are more apparent in inflammatory subgroups than in unselected samples. Modern evidence for L-tryptophan monotherapy is sparse. Trials of 5-hydroxytryptophan and interventions directed at the gut microbiota have also produced mixed results. Studies should characterize participants and sampling conditions more carefully. Diet and competition among amino acids need to be recorded. Albumin concentration, medication exposure, and disease state also affect interpretation. Considering these variables together may improve biomarker analyses and support trials in more biologically homogeneous groups. Dietary tryptophan allocation is proposed for these research purposes, not for clinical diagnosis or routine supplementation.

## 1. Introduction

Major depressive disorder (MDD) is diagnosed clinically, but patients who meet the same diagnostic criteria may differ considerably in their underlying biology. Serotonergic neurotransmission remains relevant to treatment, although available findings do not indicate the same serotonergic abnormality in all patients. Imaging studies report variable results across MDD cohorts [1]. The same applies to inflammation, as elevated inflammatory markers are found mainly in a subgroup of patients [2,3,4]. This is relevant to nutrition studies because tryptophan (Trp) availability is influenced by absorption, competition with other amino acids, host metabolism, and microbial metabolism in the gut.

Different tryptophan measures reflect different biological compartments. Total and free tryptophan, together with albumin, inform peripheral availability; the tryptophan-to-large neutral amino acid (Trp/LNAA) ratio is more relevant to competition for brain transport. Kynurenine (KYN) metabolites describe downstream host metabolism, whereas indoles arise from microbial transformations in the gut [5,6,7,8]. In a meta-analysis of 121 studies, free and total tryptophan and the Trp/LNAA ratio were lower in major depressive and bipolar disorders, while the KYN/Trp ratio was not increased. A low tryptophan concentration alone cannot be read as greater indoleamine 2,3-dioxygenase (IDO) activity [9].

Recent syntheses also fail to identify a single kynurenine profile in depression. Results vary with episode or remission, medication, inflammatory phenotype, and the matrix analyzed. A meta-analysis of cerebrospinal fluid studies found no significant difference in any measured kynurenine pathway metabolite in MDD, although several analytes were represented by only a few datasets [10,11,12,13]. Such variation is not a side issue; it is the main interpretive problem addressed here.

Earlier reviews have discussed the kynurenine pathway in depression, microbial tryptophan metabolism, and the limitations of KYN/Trp as a proxy for IDO, usually in separate syntheses [6,10,12,14]. Here, dietary supply and competition for brain transport are considered alongside host serotonin and kynurenine metabolism. Microbial indoles and clinical modifiers are then used to examine why results diverge among studies. Dietary tryptophan allocation organizes this analysis; it is not a newly proposed biochemical pathway. The same concept helps identify measurements that should accompany biomarker studies and nutrition trials.

When a cited study enrolled patients with a clinical diagnosis, depression denotes MDD. Findings based on symptom scales, experimental immune activation, animal behavior, or cell culture are identified separately. They can clarify mechanisms but cannot establish activity of a pathway or efficacy in MDD. The aim is to identify which components of the tryptophan allocation concept are supported by human evidence, which remain experimental, and how these differences should inform sampling and patient selection.

### Methodological Approach

The PubMed search included English-language records published between 1 January 1990 and 1 July 2026 and was last updated on 1 August 2026. During revision, targeted Scopus searches were conducted to identify recent reviews, meta-analyses, and human studies not retrieved through PubMed. No additional eligible publications were found. Six PubMed search blocks were used for literature on tryptophan availability and transport, kynurenine biomarkers and neurobiology, IDO and TDO regulation, inflammatory and metabolic phenotypes, omics and patient stratification, microbial metabolism, diet, safety, and serotonin imaging. After the searches were combined and duplicates removed, 5238 records remained. Of the 5238 titles, 762 were retained for abstract assessment. Abstract screening identified 145 candidate publications. The final synthesis included 114 publications.

Immune challenge experiments and preclinical studies were included only when they provided mechanistic information that could not be obtained directly from human studies. Records published before 1990, studies outside the disease scope, protocols, corrections, and reports without biological or clinical outcomes were excluded. Omics studies were also excluded when they did not report a result involving tryptophan, kynurenine, serotonin, or indole metabolites. Findings from animals and cell culture were treated as mechanistic evidence and not as evidence of efficacy or pathway activation in MDD. The structured search and screening increased transparency, but the narrative synthesis remains susceptible to literature selection bias and was not conducted as a systematic review. The exact search strings, screening criteria, and record counts are reported in Appendix A, Table A1.

## 2. Dietary Tryptophan Allocation

Dietary intake does not correspond linearly with tryptophan availability in the brain. After absorption, tryptophan circulates in free and albumin-bound pools [7]. Then it may be used in protein synthesis and metabolized in the liver. It may also enter the kynurenine pathway or serve as a precursor for serotonin and, subsequently, melatonin [15,16]. Total plasma tryptophan alone cannot distinguish substrate availability from pathway flux.

A meta-analysis of 121 articles included 15,470 participants with MDD or bipolar disorder and healthy controls. Free and total tryptophan were lower in the affective disorder groups, as was the ratio of tryptophan to competing amino acids. Kynurenine was also lower, while the kynurenine to tryptophan (KYN/Trp) ratio was unchanged or lower. The plasma difference in kynurenine was significant, but the serum and central nervous system comparisons were not. These pooled results do not isolate MDD from bipolar disorder, but they show why low tryptophan should not be equated with greater IDO activity [9].

Transport across the blood–brain barrier represents another control point in brain tryptophan availability. However, brain entry is not determined solely by plasma tryptophan levels. Boado et al. identified L-type amino acid transporter 1 (LAT1) at the blood–brain barrier and showed that other LNAAs can inhibit tryptophan transport through this system [17]. Meal composition modifies plasma amino acid availability after eating. A later controlled breakfast study confirmed that carbohydrate-rich and protein-rich meals produce distinct plasma Trp/LNAA and tyrosine-to-LNAA profiles [18]. Habitual diet was examined in 63,277 UK Biobank participants. A higher dietary ratio of tryptophan to other large neutral amino acids was associated with a modestly lower risk of depressive symptoms, and associations with variants in serotonin and kynurenine pathway genes appeared mainly in the group with a low dietary ratio. The study was observational, used estimated habitual intake, and does not show that increasing the ratio prevents or treats MDD [19].

This matters in depression research. A trial that measures plasma tryptophan without recording fasting state and meal composition risks misattributing nutritional variation to depressive biology. The same tryptophan concentration may have a different meaning in a metabolically healthy participant and in a patient with obesity or with insulin resistance. Human obesity has been associated with activation of the kynurenine pathway and a shift toward kynurenine monooxygenase activity [20]. The KYN/Trp ratio has also been associated with incident type 2 diabetes in individuals with coronary artery disease [21].

Entry of tryptophan into the central nervous system does not by itself define serotonergic output. Tryptophan hydroxylase 2 controls serotonin synthesis in the brain [22]. In humans, acute tryptophan depletion lowers cerebrospinal fluid 5-hydroxyindoleacetic acid [23], but marked mood effects occur mainly in remitted or otherwise vulnerable groups [24,25,26,27,28,29,30,31]. Precursor availability is thus better interpreted as a probe of serotonergic susceptibility than as evidence of a universal serotonin deficiency.

Kynurenine follows related transport logic. It crosses the blood–brain barrier through systems shared with LNAAs [32]. Leucine can compete with kynurenine for blood-to-brain transport in experimental inflammation [33]. Peripheral kynurenine pathway activation does not automatically define central kynurenine exposure. Blood and cerebrospinal fluid kynurenine pathway measures show relationships specific to metabolites rather than uniform correspondence [34,35,36].

Dietary tryptophan allocation can be examined from intestinal absorption through the circulating pool, competition for brain uptake, and entry into host or microbial pathways. Gut microorganisms convert luminal tryptophan into indole derivatives, some of which affect AhR and IL-22 signaling in experimental systems [14,37,38]. Table 1 presents these domains as an interpretive structure for research. It is not a validated clinical measurement panel.

## 3. Microbial Tryptophan Metabolism

The intestinal microbiota can convert luminal tryptophan into indole derivatives, whereas absorbed tryptophan enters host serotonin and kynurenine metabolism [14,37]. Some microbial indoles activate AhR and IL-22 signaling and help maintain the epithelial barrier in experimental systems [37,38]. The full sequence often proposed for depression has not been demonstrated in patients with MDD. It begins with dysbiosis and loss of indole signaling, followed by barrier disruption, cytokine release, and IDO activation. Here it is treated as a mechanistic hypothesis [44,45].

Circulating tryptophan can fall for several reasons, including diet, absorption, albumin binding, hepatic or renal handling, sample collection, and activity of host IDO or TDO. Microbial use of tryptophan may add to this variation. Neither low tryptophan nor KYN/Trp alone identifies IDO activity [6,16].

Clinical findings for microbiome interventions are mixed. *Bifidobacterium breve* CCFM1025 improved depressive and gastrointestinal symptoms in 45 patients with MDD [46]. In another trial, adding *Lactobacillus plantarum* 299v to SSRI treatment improved selected cognitive measures and lowered plasma kynurenine among 60 completers, without changing the measured cytokines or cortisol [47]. A newer study in 108 patients with MDD and gastrointestinal symptoms was negative for its primary outcome. A four-strain probiotic added to *Morinda officinalis* oligosaccharides did not improve HAMD-17 scores at week 8, although insomnia improved at week 2 [48]. Across 23 randomized trials, probiotics reduced depressive symptoms, but heterogeneity was high; the same meta-analysis found no significant benefit of prebiotics [49]. Inulin added to calorie restriction was also neutral for depression and circulating tryptophan or kynurenine measures [50]. These studies do not support a uniform class effect.

A longitudinal study followed 110 patients with MDD during 12 weeks of escitalopram treatment and included 166 healthy participants as a reference group. Integrated analysis linked the gut microbiota with plasma tryptophan and indole-3-propionic acid. At baseline, patients who later achieved remission had greater microbial richness and lower dysbiosis scores than nonremitters. A model using sporulation genes predicted remission with an AUC of 0.71. All patients received escitalopram, and the model was evaluated by nested cross-validation within the same cohort [51].

Adolescent depression has also been studied using paired microbiome and tryptophan measurements. Several genera, including *Roseburia*, were less abundant in adolescents with a first diagnosis and increased after sertraline treatment. The human component was observational. Fecal transfer and administration of *Roseburia intestinalis* in mice increased serotonin, reduced kynurenine, QUIN, and 3-HK, and improved behavior during chronic restraint stress. The mouse experiments support a possible direction of effect, but they do not establish that *Roseburia* treatment would alter depression in adolescents [52]. Larger multicohort analyses add to this evidence but are not specific to the indole branch. Zhao et al. compared 186 patients with first-episode depression and controls, then used three independent cohorts of 223, 85, and 52 participants. They identified 34 altered metabolites, 16 of which changed in the opposite direction after drug treatment. A 34-metabolite model yielded AUC values of 0.82 and 0.80 in test and validation sets. Serotonin was among the highlighted features, but the study does not show that a dietary change in tryptophan caused the metabolic pattern [53]. Cognitive findings provide another example of association without established mediation. In 86 patients with MDD and 120 controls, microbiome composition differed between groups and correlated with cognitive domains, cytokines, KYN/Trp, 3-HK, KYNA, and QUIN. Because microbiota, metabolites, cytokines, and cognition were measured within a cross-sectional design, the analysis cannot determine which component preceded the others [54].

**Figure 1:**
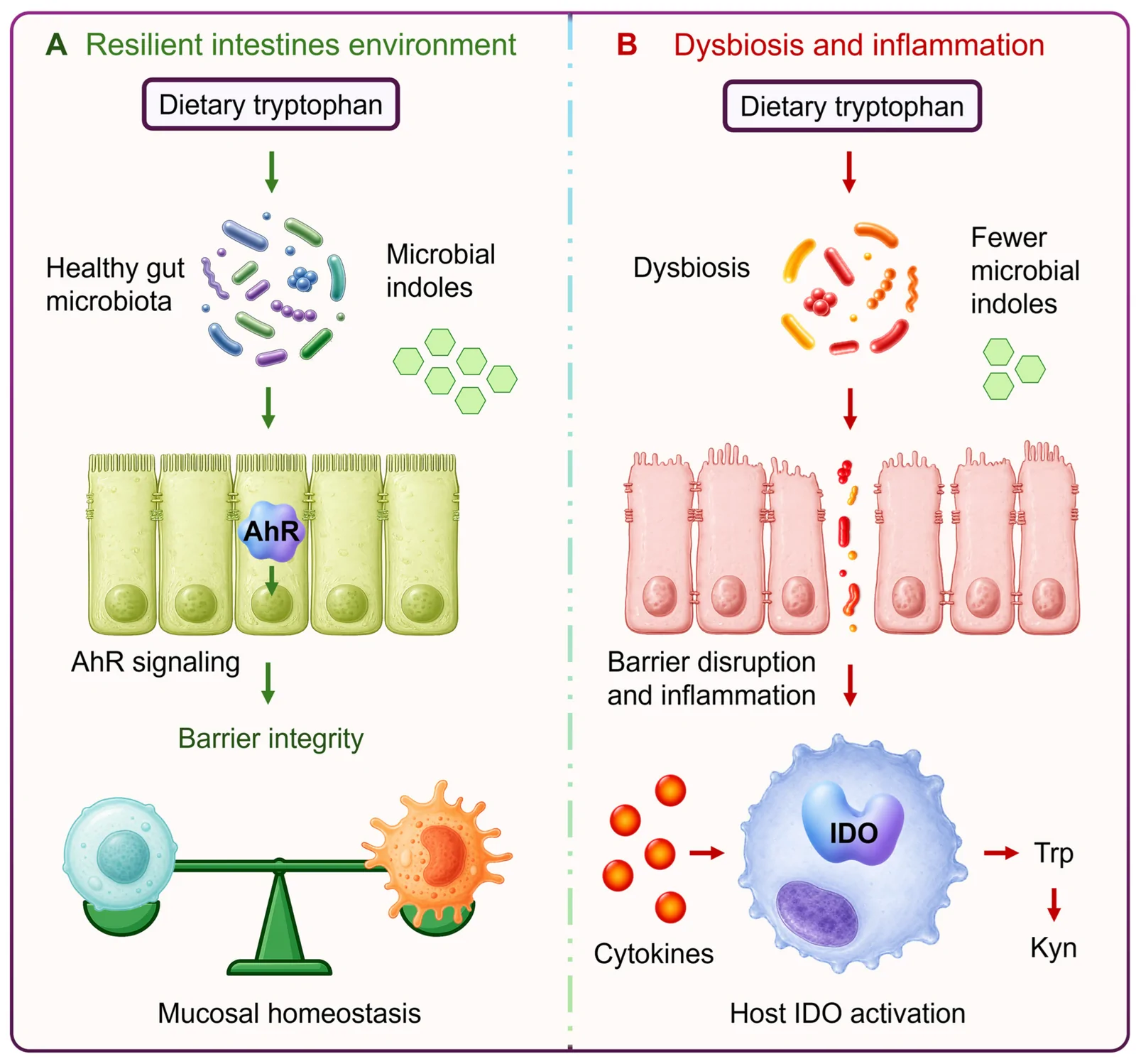
Microbial indole signaling and host kynurenine metabolism. Panel (**A**) summarizes experimentally supported AhR and IL-22 signaling by selected microbial indoles. Panel (**B**) is a conceptual model; a causal sequence from dysbiosis to barrier disruption, inflammation, IDO activation, and greater kynurenine formation has not been demonstrated in MDD. Abbreviations: AhR, aryl hydrocarbon receptor; IDO, indoleamine 2,3-dioxygenase; KYN, kynurenine; Trp, tryptophan.

Direct human evidence linking individual microbial indole derivatives with diagnosed MDD remains limited. Microbial indole pathways should thus be treated as mechanistic and translational hypotheses. Future studies should assess and interpret multiple dimensions together. Gut–brain signaling [37], alterations in tryptophan metabolism in depression [14], and activation of immune and metabolic pathways [44] can then be interpreted together. This intestinal component of tryptophan allocation is illustrated in Figure 1.

## 4. Inflammatory and Metabolic Phenotypes Shaping Kynurenine Pathway Entry

Inflammatory activation is not present in all cases of MDD. Meta-analyses report elevated CRP and selected cytokines in MDD. However, chronic low-grade inflammation appears to define only an immune-metabolic subgroup within MDD [3,4]. In a mouse model using Bacillus Calmette–Guérin, interferon gamma (IFN-γ) and tumor necrosis factor alpha (TNF-α) were required for marked IDO induction and later behavior resembling depression [55]. The cell and animal experiments establish biological plausibility, not the frequency of this mechanism in MDD.

Interferon alpha (IFN-α) treatment provides a human model of strong immune stimulation. Peripheral and CSF kynurenine increased, and CSF QUIN and KYNA rose with CSF kynurenine despite lower peripheral tryptophan and no fall in CSF tryptophan [56,57]. The findings support a change in kynurenine metabolism during immune therapy. They should not be generalized to patients who have not received an immune challenge without qualification.

Tryptophan 2,3-dioxygenase is regulated in the liver by glucocorticoids and substrate availability [16]. In a stress model, pharmacological inhibition of hepatic TDO attenuated behavior that could be attributed to depression [39]. This supports a role for TDO during experimental stress but does not establish the same mechanism in human MDD.

Clinical phenotype also matters. Among 84 patients with a current depressive episode and somatic symptoms, those with CRP ≥ 3 mg/L had more severe depression (adjusted mean difference 8.82; 95% CI, 3.91–13.72), more somatic symptoms, anxiety and perceived stress, and greater fatigue, but not greater anhedonia. The selected sample and small group size limit generalization to all MDD [58]. This result also explains why anhedonia should not be automatically assigned to every inflammatory phenotype. A metabolite study using a different CRP threshold supports biochemical heterogeneity. Data from two trials included 170 patients with MDD, of whom 127 had hs-CRP ≥ 1 mg/L and 43 had lower concentrations, as well as 80 healthy controls. QUIN was higher in the inflammatory group at *p* < 0.01, while 3-HK and kynurenine were higher at *p* < 0.05. The unequal subgroup sizes and combination of two trials make replication important [59].

IL-1 increases IDO expression caused by IFN-γ in human mononuclear phagocytes [40], while TNF-α enhances transcriptional synergy with IFN-γ, partly through NF-κB activation [41,42], and strong upregulation of IDO and depressive behavior in mice exposed to Bacillus Calmette-Guérin required IFN-γ and TNF-α [55]. Human immune challenge studies support this link. During IFN-α therapy, peripheral and CSF kynurenine both increased; CSF QUIN and KYNA rose with higher CSF kynurenine. This occurred despite a fall in peripheral tryptophan and no decrease in CSF tryptophan [57]. Increases in cerebrospinal fluid kynurenine and QUIN correlated with central immune markers and depressive symptoms. This pattern points to a shift in kynurenine metabolism instead of a simple serotonin-precursor depletion [57]. Another study reported changes in tryptophan metabolism accompanied by IFN-α and related to depression and paroxetine treatment [56]. In a stress model, pharmacological inhibition of hepatic TDO attenuated depression-like behavior, supporting a role for TDO during experimental stress without establishing the same mechanism in human MDD [39].

Metabolic phenotype deserves separate consideration. Obesity has been associated with greater kynurenine pathway activity and a shift toward kynurenine 3-monooxygenase, and in a coronary disease cohort, urinary KYN/Trp also predicted incident type 2 diabetes [20,21]. These findings support recording adiposity, insulin resistance, and liver and renal function in depression biomarker studies [6]. Table 2 summarizes these phenotype domains for research use, which have not been validated for diagnosis or treatment selection.

## 5. Serotonin–Kynurenine Balance and Brain Consequences

Acute tryptophan depletion lowers precursor availability and central serotonin metabolism. A full depressive episode is, however, uncommon in the general population, with mood effects clearest in people who are remitted or otherwise vulnerable. The experiment does not verify that low dietary tryptophan is a general cause of MDD, but tests sensitivity to precursor availability [24,25,26,28].

Serotonergic markers vary and depend on measurement methods. Molecular imaging studies do not support a single, uniform serotonergic abnormality in depression. PET studies of 5-HT1A receptors have reported lower binding in the raphe nuclei and mesiotemporal cortex, but higher binding in antidepressant-naive or remitted patients when binding potential calculated relative to free plasma radioligand concentration modelling was used [1,68,69,70,71]. SERT PET/SPECT studies are also heterogeneous, with findings varying by cohort, ligand, modelling approach, brain region, sex, season, and medication-related factors [72,73,74]. These findings show that serotonin is clinically important but argue against a single-deficit explanation.

Cell type affects downstream metabolism. Human astrocytes can produce KYNA, while IDO expression and QUIN production differ among cultured human brain cells after inflammatory stimulation [75,76,77]. Localization of kynurenine aminotransferase II has also been studied in the rat brain [78]. These experiments explain why peripheral kynurenine need not have one fixed central consequence, but cell culture and rat tissue do not quantify pathway flux in MDD.

Animal experiments show that changing endogenous KYNA can alter extracellular glutamate and performance in selected cognitive tasks [79,80]. Other models connect 3-HK, kynurenine 3-monooxygenase, oxidative stress, and behavior during inflammation [81,82,83,84,85,86,87,88]. They show that the pathway can influence neural function under experimental conditions. They do not establish an optimal KYNA concentration or the prevalence of these mechanisms in patients.

Meta-analyses indicate a pattern dependent on disease state and biological matrix. Across 101 studies and 10,912 participants, tryptophan and kynurenine were lower in MDD, bipolar disorder, and schizophrenia. KYNA and KYNA/QUIN were lower in mood disorders, while KYN/Trp was higher in MDD [12]. A later analysis of 51 studies and 7056 participants found lower tryptophan, kynurenine, KYNA, KYNA/QUIN, KYNA/3-HK, and KYNA/KYN during a current episode, with a higher KYN/Trp ratio. No significant differences were detected in remitted MDD, and the heterogeneity was high [13]. These pooled results conflict with the persistent reduction in KYNA/QUIN reported in an individual study of current and remitted MDD [43]. Persistence after remission therefore remains unresolved.

Results from cerebrospinal fluid are less consistent with peripheral activation. In a meta-analysis of 11 MDD datasets, measured kynurenine pathway metabolite did not differ significantly from controls, and only two were available both for KYNA and for QUIN [11]. Another large meta-analysis found lower tryptophan without an increase in KYN/Trp [9]. Matrix, disease state, medication exposure, albumin, and analytical methods can therefore change the apparent pathway pattern. Treatment studies add a temporal dimension. In 173 patients followed for six months, several plasma metabolites increased after antidepressant treatment, and increases in kynurenine and KYN/Trp were associated with larger reductions in HDRS scores. The direction differs from a simple model in which higher KYN/Trp must indicate worse depression. Treatment exposure and clinical improvement should therefore be analyzed together [65]. ECT does not produce a uniform biochemical pattern either. A systematic review included 17 studies, with 386 patients and 27 controls, finding no consistent change in tryptophan, kynurenines, or their ratios after treatment. The methods applied varied considerably [89].

**Figure 2:**
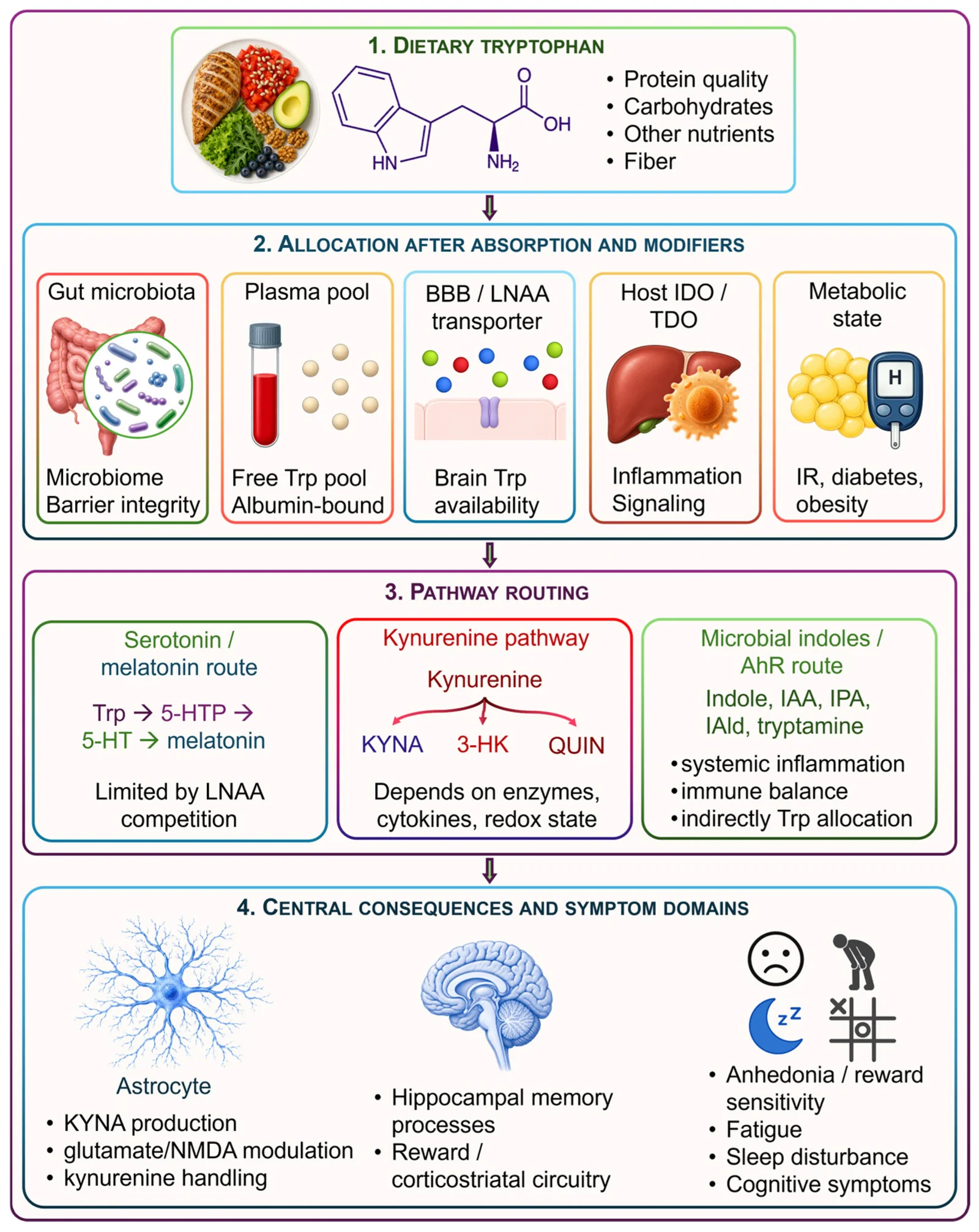
Integrated model of dietary tryptophan allocation in depression. The diagram integrates dietary supply, allocation compartments, host and microbial routes, and research endpoints. Arrows indicate proposed relationships for study design and do not denote a validated causal sequence in MDD. Abbreviations: 3-HK, 3-hydroxykynurenine; 5-HT, 5-hydroxytryptamine (serotonin); 5-HTP, 5-hydroxytryptophan; AhR, aryl hydrocarbon receptor; BBB, blood–brain barrier; IAA, indole-3-acetic acid; IAld, indole-3-aldehyde; IDO, indoleamine 2,3-dioxygenase; IPA, indole-3-propionic acid; IR, insulin resistance; KYNA, kynurenic acid; LNAA, large neutral amino acid; NMDA, *N*-methyl-D-aspartate; QUIN, quinolinic acid; TDO, tryptophan 2,3-dioxygenase; Trp, tryptophan.

A systematic review identified 22 imaging studies that related kynurenine pathway measures to the anterior cingulate and orbitofrontal cortices, striatum, thalamus, amygdala, and selected white matter tracts. The results were inconsistent, and the review called for prospective studies because the direction of the associations remained unresolved [90]. In a separate study, MDD patients (*n* = 49) and controls (*n* = 68) underwent diffusion imaging and plasma metabolomics. A metabolite module enriched for tryptophan metabolism correlated with one measure of temporal white matter connectivity, and kynurenine correlated inversely with another. The sample was small, and long-term effects of antidepressants were not excluded [91]. Neuroimaging therefore localizes associations but does not show that peripheral metabolites cause the brain findings. Figure 2 integrates dietary, post-absorptive, host, microbial, and central components of tryptophan allocation in depression.

## 6. Biomarker Interpretation, Omics Analyses, and Patient Stratification

Tryptophan and kynurenine measured in blood are peripheral markers. Their concentrations depend on diet, albumin, inflammation, metabolic state, medication, and sample handling, so they cannot be used as direct readouts of serotonin or kynurenine metabolism in the brain. Metabolomic studies have associated these measures with several features of MDD, including symptom severity, treatment resistance, risk status, and first episode disease. None of these studies established a marker suitable for diagnosis [92,93,94].

A synthesis of 143 metabolomic and 23 proteomic studies identified 11 blood metabolites that changed in a consistent direction. Tryptophan showed the highest degree of convergence. KYNA, kynurenine, and serotonin were also repeatedly lower, while only one circulating protein was consistently replicated [95]. A separate analysis used 11 public mass spectrometry datasets, with 1050 samples and 1111 unique metabolites. Changes involved tryptophan and other aromatic amino acids, as well as glutamatergic, energy, and nitrogen metabolism. No individual metabolite was established as a diagnostic marker [96]. Results for tryptophan and the early kynurenine pathway also differ between cohorts. These results support recurring metabolic disturbances in depression, while individual metabolite values remain too variable for diagnostic use.

Findings for the upper part of the kynurenine pathway are also inconsistent. Some small studies identified combinations of tryptophan, kynurenine, and lipid metabolites in first episode MDD, whereas others reported a lower KYN/TRP ratio without differences in either metabolite or found no elevation of plasma kynurenine pathway markers during an active depressive episode [97,98,99]. Meta-analyses likewise differ in the direction and magnitude of changes in KYN/TRP [9,12,13]. The ratio should not be described as a direct assay of IDO unless TDO activity, albumin, substrate availability, and downstream enzymes have been considered [6].

Metabolites from individual branches may carry more information than tryptophan or kynurenine alone. Blood values remain indirect, and their relation to central metabolism must be validated separately for each biological matrix [34,35,36].

Studies of treatment response illustrate both the potential and the current limits of stratification. In a metabolomic study with 88 patients and 88 controls, KYNA was lower in MDD. Among 62 patients who completed about six weeks of escitalopram, lower KYNA and kynurenine were associated with a larger reduction in depression scores [100]. In another study, 100 patients and 100 controls were classified by personality measures. A metabolomic classifier had an AUC of 0.715 in the whole sample and 0.907 in the cluster with less separation by personality; tryptophan, serotonin, and kynurenine were particularly low in that cluster. The stratification was derived within the same case–control dataset and requires external validation [101]. Ketamine provides another example. In treatment-resistant depression, the KYNA/QUIN ratio predicted response, while a reduction in QUIN after infusion was associated with a larger decrease in MADRS score. The same report also included an LPS mouse model, so its clinical and experimental findings should be distinguished [102].

Treatment exposure also changes the metabolome. In 163 patients randomly assigned to escitalopram, duloxetine, or cognitive behavioral therapy, the two drug groups showed lower serum serotonin and higher microbial indole derivatives after 12 weeks, whereas the psychotherapy group did not. Different metabolites correlated with improvement in each arm [64]. In a separate longitudinal cohort of 173 patients and 214 controls, KA, 3-HAA, and picolinic acid increased during six months of antidepressant treatment. Increases in kynurenine and KYN/Trp were associated with lower HDRS scores, although KYNA and KYN/Trp did not return to control levels [65]. These studies describe associations during treatment; neither validates a rule for choosing an antidepressant. The same distinction applies to cognitive phenotype. In the cross-sectional study by Zhang et al., bacterial taxa correlated with cognition, inflammatory factors, and several kynurenine pathway measures. The result supports joint measurement of these domains, but it does not justify a causal chain from microbiota through kynurenines to cognitive impairment [54].

Extracellular vesicle metabolomics has also been examined in 50 controls, 60 patients with responsive MDD, and 65 patients with treatment-resistant depression. The strongest abnormalities occurred in treatment-resistant depression. Eight weeks of duloxetine, bright-light therapy, or esketamine produced partly distinct metabolic changes in small treatment groups of 30, 30, and 35 patients [63]. The specialized matrix and absence of an independent validation cohort make this a mechanistic result, not a clinical test.

Matrix and processing time should be reported. Kynurenine pathway concentrations differed among serum, plasma, and whole blood, and several metabolites declined when blood processing was delayed for 24 h at 4 °C [103]. In depressed subjects, the link between sleep disturbance and altered kynurenine metabolism may connect sleep problems and depression [62]. Research reports should, at minimum, document dietary intake, a fasting amino acid profile with Trp/LNAA, albumin, and CRP or IL-6. Selected kynurenine metabolites, adiposity or insulin resistance, medication, sampling time, and the interval before processing would allow closer interpretation. Microbiome or central measures can be added when feasible. This is a proposed reporting set; it is not a diagnostic panel or a method for selecting treatment.

## 7. Nutrition, Supplementation, and Microbiome

L-tryptophan supplementation in depression has been considered regarding its role as a serotonin precursor. Adjunctive L-tryptophan with fluoxetine showed a clinically interesting profile. It was associated with greater HDRS-29 improvement during the first week, while polysomnography suggested protection against the reduction in slow-wave sleep observed after 4 weeks of fluoxetine treatment, without reported serotonin syndrome [104].

5-hydroxytryptophan (5-HTP), the product of tryptophan hydroxylation and a direct serotonin precursor, showed a positive response rate of 73.33%, compared with 80% for fluoxetine, in a randomized trial of first-episode depression. However, the absence of a placebo arm and the restricted patient group limit the interpretation of this finding [105]. A systematic review reported a positive effect of 5-HTP across studies, while noting their small sizes and heterogeneity [106]. Another article identified limited study quality and safety concerns [107].

α-lactalbumin is a whey protein rich in tryptophan, which a study used to increase the plasma Trp/LNAA ratio and improve mood and cortisol responses in subjects vulnerable to stress [108]. However, this effect did not clearly extend to unmedicated recovered depressed patients. The α-lactalbumin-enriched diet increased tryptophan availability but had only minimal effects on mood and cortisol responses to experimental stress [109]. These studies examined responses to laboratory stress, not treatment of an active depressive episode.

Peripheral metabolism also plays a role in modifying responses. L-tryptophan may be involved in protein synthesis or hepatic oxidation before reaching central serotonergic signaling [15,16]. 5-HTP can bypass tryptophan hydroxylase. It is, however, converted peripherally, delaying central effects [106,107]. Neither should be presented as a simple route to increased synaptic serotonin.

Inflammation may alter the response to supplementation through greater kynurenine pathway activity. This has been shown during the marked immune stimulation produced by IFN-α, not in ordinary MDD [56,57]. Whether precursor supplementation works less well in patients with an inflammatory phenotype remains to be tested.

Metabolic dysfunction may also modify supplementation outcomes. Obesity and insulin resistance can bias tryptophan metabolism toward kynurenine pathway activity [20,21]. In such phenotypes, increasing intake may not address the main driver of altered pathway balance. Low intake or an unfavorable Trp/LNAA ratio may be useful as enrollment variables in precursor trials, but neither is an established indication for supplementation [25,31,108].

Evidence is strongest for probiotics, although the trials remain heterogeneous. A meta-analysis of 23 trials involving 1401 patients found lower depressive symptom scores after probiotics, whereas the effect of prebiotics was not significant [49]. *Bifidobacterium breve* CCFM1025 improved depression and gastrointestinal scores in a four-week trial of 45 patients [46]. By contrast, adding inulin to calorie restriction for eight weeks did not change tryptophan, kynurenine, the Trp/KYN ratio, or HDRS in 51 obese women with mild MDD [50]. The effects should be reported for each preparation. Table 3 summarizes nutritional and microbiome interventions relevant to tryptophan allocation.

## 8. Safety, Translational Limitations, and Future Study Design Implications

Safety depends on precursor dose, concomitant serotonergic medication, product quality, and metabolic phenotype, while analytical handling affects how exposure and pathway markers are interpreted.

Interventions involving L-tryptophan or 5-HTP require clinical caution—both are often presented as nutritional precursors of serotonin neurotransmission. This, however, should not diminish their pharmacological relevance or safety considerations [106,107]. Excessive serotonergic activity is most often associated with combinations of serotonergic agents, including their overdose [112]. For this reason, trials inspecting precursors should record antidepressant exposure, dose changes, adverse effects, and observed symptoms that can be linked to excessive serotonergic activity. Unipolar MDD should also be distinguished from bipolar disorder or histories of mood change. The available precursor studies do not sufficiently stratify these populations [106,107].

The 1989 eosinophilia–myalgia syndrome outbreak remains an important product quality concern. More than 60 minor contaminants were identified in material from the implicated manufacturer, and six were associated with cases [113]. A 2023 review concluded, however, that experiments have not established a clear causal link between individual impurities and the syndrome [114]. Studies should transparently report manufacturer and possibly other identifying information on the source and quality of the material, as well as adverse effects.

Analytical limitations should be handled with the same specificity. Matrix, processing delay, fasting state, sampling time, albumin, medication, and recent diet can change pathway measures [62,103]. These variables should be specified in advance. Results from exploratory subgroups or panels derived in one cohort should be validated in an independent cohort before clinical interpretation.

Intervention studies need a clearer account of who was studied and how samples were obtained. At enrollment, reports should specify diagnostic state, inflammatory and metabolic phenotype, diet, Trp/LNAA, albumin, and medication exposure. Collection time and the interval before processing must accompany each biochemical outcome, together with the biological matrix. Clinical and pathway measures should be analyzed jointly. Any subgroup or biomarker panel then needs testing in an independent cohort.

## 9. Conclusions

Tryptophan availability and metabolism differ across populations with depression. Findings on kynurenine metabolism in MDD remain inconsistent. Lower peripheral tryptophan has been reported relatively often, but neither kynurenine nor its metabolites show a reproducible pattern across studies. Part of this disagreement may reflect differences in the populations examined and the laboratory procedures used. The limited evidence from cerebrospinal fluid has not clarified the relation between central and peripheral changes.

Recent studies have examined the microbiome alongside metabolite profiles. Within individual cohorts, microbial composition has been associated with tryptophan metabolites and, in some cases, with cognition or response to treatment. Most of these observations remain observational, while evidence suggesting a direction of effect still comes largely from animal experiments. Modern clinical evidence for L-tryptophan in diagnosed MDD is confined to one small augmentation trial. A brief trial of a combined supplement in people with subclinical symptoms cannot establish the efficacy of L-tryptophan alone.

The allocation model is best used to formulate research questions. It assumes that the clinical significance of tryptophan varies according to its availability and metabolism, and studies should identify patient groups that may benefit from interventions involving tryptophan. At present, the model supports patient stratification as a research hypothesis but does not justify routine treatment with L-tryptophan.

## Figures and Tables

**Table 1:** Operational meaning of dietary tryptophan allocation in depression research.

Allocation Domain	What It Reflects	Suggested Measures	Main Limitation
Dietary supply: tryptophan intake and protein intake [15,16]	Tryptophan enters the gastrointestinal system	Dietary records; estimated tryptophan intake	Intake does not equal available tryptophan
Meal context: macronutrients and Trp/LNAA [18,19]	Competition among amino acids	Meal timing, protein and carbohydrate intake	Postprandial measures and estimated habitual intake answer different questions
Circulating availability: total and free tryptophan, albumin [7,9]	Plasma tryptophan pools	Total tryptophan; free tryptophan; albumin	Values differ by matrix and do not establish central exposure or IDO activity
Host kynurenine entry: IDO and TDO context [6,9,39,40,41,42]	Oxidative tryptophan catabolism	Kynurenine; KYN/Trp	Ratio is not specific for IDO activity
Downstream pattern: KYNA, 3-HK, and QUIN [11,12,13,43]	Balance among neuroactive metabolites	KYNA; 3-HK; QUIN; KYNA/QUIN ratio	Direction differs by disease state and matrix; limited CSF evidence
Microbial use: indole pathway context [14,37,38]	Microbial tryptophan transformation	Stool profiling: microbial indoles	Microbial and host sources may overlap
Central compartment: brain readouts [34,35,36]	Central exposure and response	CSF metabolites; neuroimaging	Often unavailable in nutrition trials

Abbreviations: 3-HK, 3-hydroxykynurenine; CSF, cerebrospinal fluid; IDO, indoleamine 2,3-dioxygenase; KYN/Trp, kynurenine-to-tryptophan ratio; KYNA, kynurenic acid; LNAAs, large neutral amino acids; QUIN, quinolinic acid; TDO, tryptophan 2,3-dioxygenase; Trp/LNAA, tryptophan-to-large neutral amino acid ratio.

**Table 2:** Phenotype domains for interpreting tryptophan allocation in depression.

Phenotype Domain	Possible Indicators	Expected Relevance	Practical Implication
Inflammatory phenotype [2,3,4,58,59,60,61]	CRP; IL-6; fatigue; anhedonia	Kynurenine pathway alterations are clearer in some groups with defined CRP; symptoms are not uniform	Interpret metabolites with inflammatory markers
Metabolic phenotype [6,20,21]	Obesity; insulin resistance; dyslipidemia	May alter pathway balance	Include metabolic markers
Gut barrier and microbial indole phenotype [8,37,38,46]	Low fiber; gut symptoms; altered indoles	May change microbial tryptophan use	Pair microbiome data with metabolites
Sleep and circadian phenotype [62]	Insomnia; shift work; irregular sampling	May alter metabolite rhythms	Standardize sampling time
Medication phenotype [63,64,65,66,67]	Antidepressants; antibiotics; metabolic drugs	May alter pathways or microbiome	Stratify medication exposure
Central vulnerability phenotype [24,28,30]	Recurrent MDD; family risk; depletion sensitivity	May shape clinical response	Link markers to symptom domains

Abbreviations: CRP, C-reactive protein; IL-6, interleukin 6; MDD, major depressive disorder; Trp/LNAA, tryptophan-to-large neutral amino acid ratio.

**Table 3:** Nutritional and microbiome interventions in depression.

Intervention and Selected Evidence	Current Human Evidence	Proposed Allocation Target	Main Limitation
L-tryptophan	Fluoxetine adjunct RCT, *n* = 30: an early HDRS-29 signal and preservation of slow-wave sleep, but no later antidepressant difference [104]. A 7-day combined supplement trial in subclinical symptoms did not isolate tryptophan [110].	Low precursor availability or serotonergic vulnerability	No modern monotherapy RCT in diagnosed MDD.
5-HTP	Active-comparator trial with 60 completers reported a response similar to fluoxetine; a meta-analysis found a large pooled effect with high heterogeneity and weak designs [105,106,107].	Possible precursor-sensitive phenotype	No placebo in the direct comparison; no validated phenotype.
α-Lactalbumin	Raised Trp/LNAA and altered stress responses in vulnerable healthy participants; minimal mood effects in recovered unmedicated patients [108,109].	Low Trp/LNAA or stress vulnerability	Laboratory stress studies, not treatment of an active episode.
Diet quality	A recent review supports diet as an adjunct approach to depression through multiple mechanisms [111].	Poor diet quality or metabolic risk	Effects are not specific to tryptophan allocation.
Prebiotics	Inulin trial, *n* = 51: no between-group effect on Trp, KYN, Trp/KYN, or HDRS; three prebiotic depression trials in a meta-analysis showed no significant pooled effect [49,50].	Low fiber intake or gut phenotype	Few trials; the direct biomarker study included calorie restriction.
Probiotics	Meta-analysis: reduction in depressive symptoms, with high heterogeneity. Direct trials found improvement in depressive and gastrointestinal symptoms with *B. breve* CCFM1025, improvement in selected cognitive measures and lower KYN with *L. plantarum* 299v, and no improvement in HAMD-17 after adding a four-strain probiotic to *Morinda officinalis* oligosaccharides [46,47,48,49,50].	Gut symptoms or a microbiome subgroup	Effects differ between strains and interventions. Symptom or cognitive changes do not establish mediation by tryptophan metabolism.
Omega-3 fatty acids	Combined analysis of two MDD trials found lower QUIN and 3-HK in an inflammatory subgroup [59].	MDD with elevated CRP and kynurenine metabolites	Requires a confirmatory trial.

Abbreviations: 3-HK, 3-hydroxykynurenine; 5-HTP, 5-hydroxytryptophan; CRP, C-reactive protein; HAMD-17, 17-item Hamilton Depression Rating Scale; HDRS-29, 29-item Hamilton Depression Rating Scale; KYN, kynurenine; LNAA, large neutral amino acids; MDD, major depressive disorder; QUIN, quinolinic acid; RCT, randomized controlled trial; Trp, tryptophan; Trp/KYN, tryptophan-to-kynurenine ratio; Trp/LNAA, tryptophan-to-large neutral amino acid ratio.

## Data Availability

No new data were created or analyzed in this study. Data sharing is not applicable to this article.

## Abbreviations

The following abbreviations are used in this manuscript:

3-HAA	3-hydroxyanthranilic acid
3-HK	3-hydroxykynurenine
5-HT1A	5-hydroxytryptamine 1A receptor
5-HTP	5-hydroxytryptophan
AhR	Aryl hydrocarbon receptor
BBB	Blood–brain barrier
CRP	C-reactive protein
CSF	Cerebrospinal fluid
HDRS	Hamilton Depression Rating Scale
IDO	Indoleamine 2,3-dioxygenase
IFN	Interferon
IL	Interleukin
KYN	Kynurenine
KYN/Trp	Kynurenine-to-tryptophan ratio
KYNA	Kynurenic acid
LAT1	L-type amino acid transporter 1
LNAA	Large neutral amino acid
LNAAs	Large neutral amino acids
MDD	Major depressive disorder
NF-κB	Nuclear factor kappa B
NMDA	*N*-methyl-D-aspartate
PET	Positron emission tomography
QUIN	Quinolinic acid
SERT	Serotonin transporter
SPECT	Single-photon emission computed tomography
TDO	Tryptophan 2,3-dioxygenase
TNF-α	Tumor necrosis factor alpha
Trp	Tryptophan
Trp/LNAA	Tryptophan-to-large neutral amino acid ratio

## Appendix A

The search strategy used to support this narrative review is reported in Table A1.

**Table A1:** PubMed search blocks and record counts used.

Search Block, Topic; Records Retrieved	Exact PubMed Query
S1. Tryptophan, Serotonin, and Precursor Availability; *n* = 642	((depletion[ti] AND tryptophan[ti] AND (depress*[ti] OR mood[ti] OR remission[ti] OR remitted[ti] OR antidepres*[ti] OR fluoxetine[ti] OR desipramine[ti] OR cognitive[ti] OR neural[ti] OR emotional[ti])) OR (tryptophan[ti] AND remission[ti] AND depression[ti]) OR (tryptophan[ti] AND depress*[ti] AND (fluoxetine[ti] OR amitriptyline[ti] OR imipramine[ti] OR nicotinamide[ti] OR “general practice”[ti] OR placebo[ti])) OR (“5-hydroxytryptophan”[ti] AND depress*[ti]) OR (“5-HTP”[ti] AND depress*[ti]) OR (tryptophan[ti] AND “large neutral amino acid”[ti]) OR (tryptophan[ti] AND “large neutral amino acids”[ti]) OR (tryptophan[ti] AND “blood-brain barrier”[ti]) OR (tryptophan[ti] AND “free tryptophan”[ti]) OR (tryptophan[ti] AND “albumin-bound”[ti]) OR (tryptophan[ti] AND brain[ti] AND (uptake[ti] OR influx[ti] OR probenecid[ti] OR insulin[ti] OR serotonin[ti])) OR (serotonin[ti] AND brain[ti] AND (“plasma tryptophan”[ti] OR “neutral amino acid”[ti] OR “neutral amino acids”[ti] OR content[ti] OR synthesis[ti])) OR (tryptophan[ti] AND “5-hydroxyindoleacetic acid”[ti]) OR (tryptophan[ti] AND “5-HIAA”[ti]) OR (“alpha-lactalbumin”[ti] AND (tryptophan[ti] OR mood[ti] OR cortisol[ti])) OR ((breakfast[ti] OR meal[ti] OR meals[ti]) AND tryptophan[ti] AND (“large neutral amino acid”[ti] OR “large neutral amino acids”[ti] OR ratio[ti] OR ratios[ti])) OR (“tryptophan hydroxylase”[ti] AND serotonin[ti]) OR (“tryptophan hydroxylase-2”[ti] AND serotonin[ti]) OR (“tryptophan hydroxylase 2”[ti] AND serotonin[ti]) OR (“L-Tryptophan”[ti] AND (therapeutic[ti] OR behavioral[ti] OR metabolic[ti])) OR (“plasma amino acid responses”[ti] AND meals[ti]) OR (“large neutral amino acid transporter”[ti] AND “blood-brain barrier”[ti]) OR (amine[ti] AND precursors[ti] AND depression[ti]) OR (tryptophan[ti] AND depletion[ti] AND CSF[ti])) AND 1990/01/01:2026/07/01[dp] AND english[la]
S2. Clinical Kynurenine Biomarkers in Depression; *n* = 893	((kynurenine[ti] OR kynurenines[ti] OR kynurenic[ti] OR quinolinic[ti] OR “kynurenine pathway”[ti] OR “kynurenine to tryptophan”[ti] OR “kynurenine-to-tryptophan”[ti] OR “tryptophan-kynurenine”[ti]) AND (depress*[ti] OR “major depressive disorder”[ti] OR “major depression”[ti] OR MDD[ti] OR biomarker*[ti] OR metabolite*[ti] OR metabolomic*[ti] OR plasma[ti] OR serum[ti] OR CSF[ti] OR sleep[ti] OR obesity[ti] OR diabetes[ti] OR ketamine[ti] OR suicidal[ti] OR “treatment resistant”[ti] OR “treatment-resistant”[ti]) OR (“plasma metabolites”[ti] AND depression[ti] AND suicidal[ti]) OR (plasma[ti] AND biomarkers[ti] AND “major depressive disorder”[ti]) OR (metabolic[ti] AND features[ti] AND “major depressive disorder”[ti])) AND 1990/01/01:2026/07/01[dp] AND english[la]
S3. Mechanistic Kynurenine Neurobiology and Toxicity; *n* = 775	((kynurenic[ti] OR kynurenate[ti] OR quinolinic[ti] OR “3-hydroxykynurenine”[ti] OR “3-hydroxyanthranilic”[ti] OR “kynurenine 3-monooxygenase”[ti] OR “kynurenine 3-mono-oxygenase”[ti] OR “kynurenine aminotransferase”[ti]) AND (brain[ti] OR CNS[ti] OR astrocyt*[ti] OR microglia*[ti] OR neuron*[ti] OR neuronal[ti] OR hippocamp*[ti] OR “basal ganglia”[ti] OR NMDA[ti] OR glutamate[ti] OR neurotox*[ti] OR cytotoxicity[ti] OR “cell death”[ti] OR “hydrogen peroxide”[ti] OR memory[ti] OR seizure*[ti] OR receptor*[ti]) OR (“kynurenine pathway”[ti] AND tryptophan[ti] AND metabolism[ti]) OR (kynurenine[ti] AND pathway[ti] AND astrocyt*[ti]) OR (kynurenines[ti] AND “blood-brain barrier”[ti]) OR (quinolinic[ti] AND excitotoxic[ti])) AND 1990/01/01:2026/07/01[dp] AND english[la]
S4. IDO and TDO Activation and Immune Signaling; *n* = 647	((“indoleamine 2,3-dioxygenase”[ti] OR “indoleamine dioxygenase”[ti] OR IDO[ti] OR “tryptophan 2,3-dioxygenase”[ti] OR TDO[ti]) AND (interferon[ti] OR cytokine*[ti] OR “tumor necrosis factor”[ti] OR TNF[ti] OR “NF-kappa”[ti] OR interleukin[ti] OR glucocorticoid*[ti] OR stress[ti] OR depress*[ti] OR behavior[ti] OR behaviour[ti] OR lipopolysaccharide[ti] OR tryptophan[ti] OR kynurenine[ti]) OR (interferon[ti] AND alpha[ti] AND tryptophan[ti] AND metabolism[ti])) AND 1990/01/01:2026/07/01[dp] AND english[la]
S5. Inflammatory and Metabolic Phenotypes, Omics Analyses, and Patient Stratification; *n* = 709	((“low-grade inflammation”[ti] AND depression[ti]) OR (“C-reactive protein”[ti] AND (“major depressive disorder”[ti] OR depression[ti] OR depressive[ti])) OR (interleukin*[ti] AND “C-reactive protein”[ti] AND depress*[ti]) OR (cytokine*[ti] AND (“major depression”[ti] OR “major depressive disorder”[ti]) AND (alterations[ti] OR meta-analysis[ti] OR patients[ti])) OR (Th1[ti] AND Th2[ti] AND Th3[ti] AND depression[ti]) OR (“serum interleukin-6”[ti] AND “C-reactive protein”[ti] AND “major depressive disorder”[ti]) OR ((“tumor necrosis factor”[ti] OR “tumour necrosis factor”[ti] OR infliximab[ti]) AND (“treatment-resistant depression”[ti] OR “treatment resistant depression”[ti] OR depress*[ti])) OR (inflammation[ti] AND corticostriatal[ti] AND depression[ti]) OR (inflammation[ti] AND anhedonia[ti] AND reward[ti]) OR (“ventral striatum”[ti] AND reward[ti] AND endotoxin[ti]) OR ((“basal ganglia”[ti] AND glutamate[ti] AND depression[ti]) OR (“basal ganglia”[ti] AND fatigue[ti] AND interferon[ti]) OR (dopaminergic[ti] AND reward[ti] AND interferon[ti]) OR (“effort-based decision-making”[ti] AND “major depressive disorder”[ti]) OR (“advanced glycation”[ti] AND “NF-kappa”[ti]) OR (“advanced glycation end product”[ti] AND “NF-kappaB”[ti]) OR (“skin autofluorescence”[ti] AND melancholic[ti]) OR (obesity[ti] AND “kynurenine pathway”[ti]) OR (“kynurenine to tryptophan ratio”[ti] AND diabetes[ti]) OR (“inflammatory biomarker”[ti] AND escitalopram[ti] AND nortriptyline[ti]) OR (biomarkers[ti] AND paroxetine[ti] AND venlafaxine[ti]) OR (“pro inflammatory markers”[ti] AND pharmacotherapy[ti] AND “major depressive disorder”[ti]) OR (“sleep disturbance”[ti] AND kynurenine[ti] AND depression[ti]) OR (“sleep deprivation”[ti] AND metabolome[ti]) OR (circadian[ti] AND metabolite[ti] AND rhythms[ti])) OR ((depress*[ti] OR “major depressive disorder”[ti] OR “major depression”[ti] OR MDD[ti]) AND (multiomic*[ti] OR “multi-omics”[ti] OR omics[ti] OR metabolom*[ti] OR proteom*[ti] OR transcriptom*[ti] OR lipidom*[ti] OR “metabolic profiling”[ti] OR “metabolic phenotyping”[ti] OR phenotyp*[ti] OR stratif*[ti] OR cluster*[ti] OR subgroup*[ti] OR “precision psychiatry”[ti] OR “precision medicine”[ti] OR “treatment prediction”[ti] OR “response prediction”[ti]) AND (tryptophan[tiab] OR kynurenin*[tiab] OR kynurenic[tiab] OR quinolinic[tiab] OR serotonin[tiab] OR indole*[tiab]))) AND 1990/01/01:2026/07/01[dp] AND english[la]
S6. Microbiome, Diet, Safety, and Serotonin Imaging; *n* = 1705	((tryptophan[ti] AND microbiota[ti] AND metabolism[ti]) OR (tryptophan[ti] AND microbiome[ti] AND (“gut-brain”[ti] OR “gut brain”[ti] OR depression[ti] OR disease[ti])) OR (microbial[ti] AND tryptophan[ti] AND catabolites[ti]) OR (tryptophan[ti] AND metabolites[ti] AND depression[ti] AND microbiota[ti]) OR (indole*[ti] AND microbiota[ti] AND (tryptophan[ti] OR “aryl hydrocarbon receptor”[ti] OR AhR[ti] OR mucosal[ti] OR intestinal[ti])) OR (“aryl hydrocarbon receptor”[ti] AND tryptophan[ti] AND microbiota[ti]) OR (“interleukin-22”[ti] AND tryptophan[ti] AND microbiota[ti]) OR (“IL-22”[ti] AND tryptophan[ti] AND microbiota[ti]) OR (anthranilic[ti] AND tryptophan[ti] AND (brain[ti] OR neurological[ti] OR disease[ti])) OR (Bifidobacterium[ti] AND depress*[ti]) OR (probiotic*[ti] AND depress*[ti] AND (trial[ti] OR review[ti] OR “meta-analysis”[ti] OR symptoms[ti] OR patients[ti])) OR (prebiotic*[ti] AND depress*[ti] AND (trial[ti] OR review[ti] OR “meta-analysis”[ti] OR symptoms[ti] OR patients[ti])) OR (inulin[ti] AND depress*[ti]) OR (“diet quality”[ti] AND depress*[ti]) OR (“diet intervention”[ti] AND depress*[ti]) OR (“diet interventions”[ti] AND depress*[ti]) OR (“nutritional psychiatry”[ti] AND depress*[ti]) OR (“serotonin transporter”[ti] AND depress*[ti] AND (binding[ti] OR PET[ti] OR SPECT[ti])) OR (“serotonin transporters”[ti] AND “major depression”[ti]) OR (“serotonin 1A”[ti] AND depress*[ti] AND binding[ti]) OR (“serotonin-1A”[ti] AND depress*[ti] AND binding[ti]) OR (“serotonin-1A”[ti] AND depression[ti] AND imaging[ti]) OR (“5-HT1A”[ti] AND depress*[ti] AND binding[ti]) OR (serotonin[ti] AND transporter[ti] AND occupancy[ti]) OR (serotonin[ti] AND transporter[ti] AND binding[ti] AND “mood disorders”[ti]) OR “serotonin syndrome”[ti] OR (tryptophan[ti] AND “eosinophilia-myalgia”[ti]) OR (tryptophan[ti] AND “eosinophilia myalgia”[ti]) OR (tryptophan[ti] AND contaminant*[ti]) OR (“kynurenine pathway metabolites”[ti] AND stability[ti]) OR (“kynurenine pathway metabolites”[ti] AND “blood processing”[ti]) OR (“kynurenine pathway metabolites”[ti] AND “blood components”[ti])) AND 1990/01/01:2026/07/01[dp] AND english[la]
Final combined search; *n* = 5238	S1 OR S2 OR S3 OR S4 OR S5 OR S6
Records selected after title screening *n* = 762	Titles were retained when they indicated direct relevance to depression and tryptophan metabolism or clear mechanistic relevance to the interpretation of serotonin synthesis, the kynurenine pathway, microbial indoles, inflammation, nutritional interventions, biomarkers, or safety. Titles concerning unrelated diseases, populations, or models were excluded unless they indicated a clear mechanistic contribution to the review. Protocols, corrections, and retracted records were also excluded.
Records selected after abstract screening *n* = 145	Abstracts were retained when they reported clinical or mechanistic findings relevant to depression and at least one of the review topics listed above. Records were excluded when the apparent relevance was not confirmed, the association with depression or tryptophan metabolism was incidental, or no usable biological or clinical result was reported.
Included articles; *n* = 114	Candidate publications were included when they provided direct clinical evidence, a recent systematic or quantitative synthesis, or mechanistic evidence needed to interpret the review. Priority was given to human studies of MDD and higher-level clinical evidence. Older landmark reports, experimental immune challenges, and preclinical studies were retained only when they addressed a foundational finding or a mechanism that could not be evaluated directly in humans.

Note: The final PubMed search was performed on 1 August 2026 and covered publications in English from 1 January 1990 through 1 July 2026. Title and abstract screening was performed by B.K. Screening decisions were reviewed by the same author on the following day against the criteria reported above. Records that remained uncertain were retained for the next stage. Targeted supplementary searches in Scopus were conducted during revision to identify recent reviews, meta-analyses, and human studies not retrieved through PubMed. No additional eligible publications were identified. The Scopus searches were not included in the PubMed record counts.
